# C-857-04. Caring for Caregivers: System-level Solutions to Moral Injury in Burn Teams

**DOI:** 10.1093/jbcr/irag033.104

**Published:** 2026-04-06

**Authors:** Joshua Khorsandi, Justin Kahen, Liahm Blank, Abu-Bakr Ahmed, Demitri Franzoni

**Affiliations:** Kirk Kerkorian School of Medicine at University of Nevada, Las Vegas, NV; Kirk Kerkorian School of Medicine at University of Nevada, Las Vegas, NV; Kirk Kerkorian School of Medicine at University of Nevada, Las Vegas, NV; Kirk Kerkorian School of Medicine at University of Nevada, Las Vegas, NV; Department of Plastic and Reconstructive Surgery, University of Nevada, Las Vegas, NV

## Abstract

**Introduction:**

Burn care depends on an interprofessional “hidden workforce” of nurses, residents, and rehabilitation therapists whose proximity to trauma, disfigurement, and high-stakes decisions creates distinctive risks for moral injury and compassion fatigue. Literature describes moral distress in acute and critical care, yet comparatively few papers frame mitigation as an institutional ethical duty rather than an individual resilience challenge. This structured review synthesizes evidence on antecedents, consequences, and system-level responses, arguing for organizational accountability in burn care.

**Methods:**

Using a predefined protocol and PRISMA-informed approach, peer-reviewed studies in English were identified across PubMed, CINAHL, PsycINFO, and Embase. Search terms combined moral injury, distress, compassion fatigue, burnout, ethics consultation, organizational ethics, and burn/critical care. Inclusion prioritized studies and reviews on nurses, physicians/trainees, and therapists in inpatient burn or analogous high-acuity settings. Data were extracted on precipitating factors, outcomes, and institutional interventions, including ethics consultation.

**Results:**

Across heterogeneous designs, recurrent antecedents included resource scarcity, triage constraints, perceived futility, repeated exposure to suffering, role conflict, and unclear authority lines. Outcomes encompassed emotional exhaustion, diminished empathy, turnover intention, and weakened team cohesion; several studies linked distress to safety and quality concerns. Protective elements most associated with improvement were multi-level measures: supportive leadership, structured peer support and debriefings, psychologically safe communication, clear escalation pathways, and timely ethics consultation. Resilience training alone was described as insufficient when unaccompanied by workload, staffing, and policy reforms. Few studies analyzed moral injury prevention as an institution’s ethical obligation or evaluated ethics consultation as a proactive (not just reactive) resource.

**Conclusions:**

Moral injury and compassion fatigue among burn care staff are organizational ethics issues. Addressing them requires moving beyond individual coping to accountable, system-level design that reduces preventable distress and normalizes early ethics input.

**Applicability to Practice:**

Burn centers can operationalize duty of care by establishing rapid-access ethics consultation, integrating routine moral distress rounds, aligning staffing and scheduling with trauma exposure, embedding validated distress measures into quality dashboards, training leaders to recognize warning signs, and ensuring protected time for debriefing after high-moral-load events. Institutions should evaluate impact with staff outcomes and patient-safety indicators.

